# Supplementary material for: What is best practice in sex and relationship education? A synthesis of evidence, including stakeholders’ views
Source: BMJ Open. 2017 Jul 2;7(5):e014791. doi: 10.1136/bmjopen-2016-014791 (PMC5730004; doi:10.1136/bmjopen-2016-014791)
Supplement: Supplementary Appendix 1 [file bmjopen-2016-014791supp001.pdf]

Appendix 1

Summary of findings from individual work packages

**Work package**	**Methods**	**Summary of findings**	**Publication**
1. Telephone interview study	Qualitative telephone interview study covering all English regions using in-depth semi-structured interviews. Sample obtained using purposive and snowball sampling. Data analysed using 'framework' method.	Forty seven individuals were interviewed, with representation from every English region.<br><br>There was strong consensus on the features to aim for in Personal, Social and Health Education (PSHE) and widespread concern about variations and gaps in current PSHE delivery and lack of statutory PSHE.<br><br>There were many examples of energetic advocacy and of continued, voluntary, engagement with the Healthy Schools programme and the legacy of the Teenage Pregnancy Strategy.<br><br>Many participants argued that effective PSHE can and should make a positive impact on attainment and attendance, but observed that it was difficult to evidence this.	In preparation
2. Qualitative synthesis	Synthesis of qualitative studies of young people's views of their sex and relationship education. Studies were identified using electronic and hand searching methods. References were double screened and eligible papers were appraised for quality by 2 independent reviewers. Data were analysed using a combination of meta-ethnography and thematic synthesis.	Sixty nine publications were identified, with 55 remaining after quality appraisal, representing 48 studies.<br><br>The synthesis found that although sex is a potent and potentially embarrassing topic, schools appeared reluctant to acknowledge this and attempted to teach SRE in the same way as other subjects. Young people reported feeling vulnerable in SRE, with young men anxious to conceal sexual ignorance and young women risking sexual harassment if they participate.<br><br>Schools appeared to have difficulty accepting that some young people are sexually active, leading to SRE that was out of touch with many young people's lives. Young people report that SRE can be negative, gendered and heterosexist.<br><br>Young people expressed dislike of their own teachers delivering SRE due to blurred boundaries, lack of anonymity, embarrassment and poor training.	Pound P, Langford R, Campbell R. What do young people think about their school-based sex and relationship education? A qualitative synthesis of young people's views and experiences. BMJ Open 2016;6(9):e011329.
3. Case studies	Three case studies selected, each comprising a distinct model of behaviour change. Data on each case were collected using documentary analysis, interviews, focus groups, informal discussions with key partners and observation of intervention delivery. Data were analysed using cross case analysis.	The 3 case studies were a social norms approach, a curriculum based risk and resilience model and a comprehensive, school-based peer education programme provided by a third sector organisation. Key factors were identified that appeared to contribute to intervention success.<br><br>Those most closely involved with delivery of sexual health interventions saw the need for a holistic, whole school approach in	In preparation

		order to encourage the intervention to become deeply ingrained in all aspects of school culture.<br><br>In practice and perhaps counterintuitively, this was easier to implement with the peer education model delivered by external agents. School-led approaches consistently emphasised negative attitudes towards sexual activity, making it difficult for young people to discuss issues freely. Teachers' poor levels of training and lack of assurance led to young people having little confidence in school-led approaches.	
4. Natsal analyses	i. Natsal-3 is a multi-stage, clustered and stratified probability sample survey of 15,162 men and women aged 16-74 years resident in Britain. Analysis explored associations between sources of information about sex and sexual health outcomes.	i. Relative to other sources, citing school was associated with older age at first sex, lower likelihood of unsafe sex and previous STI diagnosis and, in women, with lower likelihood of lack of sexual competence at first sex; and experience of non-volitional sex, abortion and distress about sex. Citing a parent was associated with lower likelihood of unsafe sex and, in women, previous STI diagnosis.	i. Macdowall W, Jones KG, Tanton C, et al. Associations between source of information about sex and sexual health outcomes in Britain: findings from the third National Survey of Sexual Attitudes and Lifestyles (Natsal-3). BMJ Open 2015;**5**(3):e007837.
	ii. The Natsal probability sample surveys to date have been carried out approximately decennially in 1990-91 (Natsal-1),23,24 1999-2001 (Natsal-2)25,26 and 2010-2012 (Natsal-3). Analysis explored trends in sources of information about sex among young people.	ii. Between 1990 and 2012, the proportion citing school lessons as their main source of information about sexual matters increased from 28.2% to 40.3%. In 2010–2012, parents were reported as a main source by only 7.1% of men and 14.1% of women and, for women, were less commonly reported than in 1999–2001.<br><br>Most young people reported not knowing enough when they first felt ready for sexual experience and this did not change substantially over time. They wanted more information about psychosexual matters, sexually transmitted infections and contraception (women). Young people primarily wanted information from school, parents or health professionals.	ii. Tanton C, Jones KG, Macdowall W, et al. Patterns and trends in sources of information about sex among young people in Britain: evidence from three National Surveys of Sexual Attitudes and Lifestyles. BMJ Open 2015;**5**(3):e007834.
5. Review of reviews	Systematic review of systematic reviews of school-based interventions to improve sexual-health. Studies were identified using electronic and hand searches. An independent reviewer checked a random sample of titles and abstracts as well as full papers. Review quality was assessed using AMSTAR. Findings were tabulated with a quality assessment of the evidence provided. A narrative synthesis was conducted.	Thirty-seven systematic reviews (summarising 224 primary randomised controlled trials) met our inclusion and quality assessment criteria.<br><br>Integration of review findings generated a list of 32 design, content and implementation characteristics that may enhance the effectiveness of school-based, sexual-health interventions.<br><br>Abstinence-only interventions were found to be ineffective in promoting positive changes in sexual behaviour. Comprehensive interventions, those specifically targeting HIV prevention, and school-based clinics were found to be effective in improving knowledge and changing attitudes, behaviours and health-relevant outcomes.	Denford S, Abraham C, Campbell R, Busse H. A comprehensive review of reviews of school-based interventions to improve sexual-health. Health Psychology Review. 2016; 11: 1-20.
